# A Comprehensive Examination of the Nature, Frequency, and Context of Parental Weight Communication: Perspectives of Parents and Adolescents

**DOI:** 10.3390/nu14081562

**Published:** 2022-04-08

**Authors:** Rebecca M. Puhl, Leah M. Lessard, Gary D. Foster, Michelle I. Cardel

**Affiliations:** 1 Department of Human Development & Family Sciences, University of Connecticut, Storrs, CT 06269, USA; 2 Rudd Center for Food Policy & Health, University of Connecticut, Hartford, CT 06103, USA; leah.lessard@uconn.edu; 3 Center for Weight and Eating Disorders, Department of Psychiatry, Perelman School of Medicine, University of Pennsylvania, Philadelphia, PA 19104, USA; gary.foster@ww.com; 4 WW International, Inc., New York, NY 10010, USA; michelle.cardel@ww.com; 5 Department of Clinical and Health Psychology, University of Florida, Gainesville, FL 32611, USA

**Keywords:** weight-talk, body weight, adolescent, parent, communication

## Abstract

Research suggests that many parents make comments about their child’s weight, which is associated with negative adolescent health outcomes. Gaps in this literature include an underrepresentation of fathers, limited knowledge regarding positive versus negative parental weight comments and differences across race/ethnicity, and adolescent preferences for parental weight communication. The present study addressed these research gaps through a comprehensive investigation of two diverse samples of U.S. parents (*n* = 1936) and adolescents (*n* = 2032), who completed questionnaires about their experiences and perspectives of parental weight communication. Positive weight comments from parents were more frequent than negative comments, though both were commonly reported across sex, race/ethnicity, and weight status. In general, boys, fathers, Latino/a parents and adolescents, and adolescents with a high BMI and/or engaged in weight management reported more frequent parental weight-talk. Parent–adolescent weight communication occurred both in-person and digitally, and across daily life contexts. Although the majority of parents communicated positive messages of body diversity and respect, 44% and 63% of adolescents said they never want their mothers and fathers, respectively, to talk about their weight. Adolescents were offered circumstances that would increase their comfort level in having these conversations. Findings have implications for health professionals working with families to promote supportive health communication at home.

## 1. Introduction

Adolescence is a period marked by important physical and psychological changes, and is a critical time for developing a positive body image [1]. Thus, throughout early, middle, and late adolescence, body weight can be an emotionally charged and sensitive topic, particularly in the context of parent–child communication [2,3]. Research suggests that talking about body weight is a common parental practice. The prevalence of adolescent-reported parental comments about weight is as high as 78% [4], although there is a broad range of this and prevalence rates can differ between girls and boys, depending on whether comments come from mothers or fathers [5,6,7]. For example, evidence suggests that parental comments about weight are typically more common among same-sex parent–child dyads [4] and more frequent for girls compared to boys [4,8]. Furthermore, given the high prevalence rates of obesity in adolescents [9] and adults [10], studies have documented a higher prevalence of weight comments among parents with adolescents who have higher body mass index (BMI) percentiles (e.g., those with overweight or obesity) [5,11,12]. Nevertheless, recent qualitative evidence with racially/ethnically diverse families suggests that parents both with and without a child with overweight/obesity engage in weight-focused conversations at home [13], highlighting the importance of studying parent weight communication in all families and not just those who have a higher body weight. Collectively, this literature indicates the presence of parental weight communication in many families.

Parental weight-focused conversations, or ‘weight-talk’, can include comments parents make about weight or shape directed toward their child, themselves, or others. Most research to date has studied negative comments that parents make about their child’s weight, shape, or size, such as critical weight comments, weight teasing, and encouraging their child to lose weight [14,15,16,17]. Evidence spanning two decades documents concerning implications of parent weight-talk for the health and wellbeing of youth [18,19,20]. A 2016 meta-analysis of 38 studies documented consistent associations between parent weight-focused conversations and negative outcomes in youth and adolescents, including psychological distress (e.g., symptoms of depression, poor body image, low self-esteem), dieting, unhealthy weight control practices (e.g., binge eating, taking diet pills), and overweight/obesity [21]. Associations were present for children of all ages, with stronger associations with dysfunctional eating for girls compared to boys. These consistent negative associations were replicated in a 2021 systematic review of the literature, focusing on adolescent health implications of parent weight-talk, showing that weight-talk is linked with adverse physical, social, and psychological consequences for adolescents, including body dissatisfaction, unhealthy weight control behaviors, higher BMI, and feelings of shame [17]. Longitudinal studies further suggest that parental weight-talk tends to persist over time, and that negative outcomes from weight-focused conversations may continue beyond adolescence into adulthood [22,23,24,25]. Taken together, this evidence underscores the potential harms of parent weight communication for the health and wellbeing of youth and adolescents.

### 1.1. Unanswered Questions and Key Gaps in Research

To date, the literature examining parent weight-talk has provided important insights, particularly with respect to youth psychosocial and health outcomes. However, many important research questions are unanswered and key gaps remain in this field of study. In their 2021 systematic review of the parent–adolescent weight-talk literature over the past decade, Yourell and colleagues identified a number of methodological shortcomings in this literature, notably the underrepresentation of fathers in existing research, the lack of knowledge about adolescent preferences and perceptions of weight communication with parents, and the need for studies to examine different types of weight-talk between parents and youth [17]. For example, most studies to date have assessed either negative parental weight-talk or general ‘comments about weight’ [14,26], without examination or consideration of positive comments that parents may engage in about weight. This limitation in particular has been acknowledged by scholars for more than a decade [20], and researchers in the field continue to note that studies have focused more on identifying consequences of weight-talk rather than on attempting to understand the context, circumstances, and nature of these conversations [13]. Thus, despite concerning health implications of parent weight-talk, well documented across studies, we know considerably less about how parents and adolescents communicate about weight. Although qualitative research has begun to highlight that parents engage in different types of weight-focused conversations [13], a comprehensive understanding of the nature and contexts of parent weight talk with adolescents has not yet been established. Collectively, these significant gaps in knowledge highlight clear priorities for research, which serve as key aims for the present study and are further described below.

### 1.2. Positive Comments and Weight Socialization

With most research, attention to date has focused on the negative aspects of parental weight communication and little is known about the prevalence and nature of positive weight-talk between parents and their children. In a recent Australian study, adolescents reported both positive and negative comments about their weight from parents, both of which were more frequent among girls (compared to boys), and from mothers (versus fathers) [4]. However, assessment of parental weight-talk in this study (using two survey questions “how often does your mother (father) comment positively (negatively) on your weight or shape?”) did not examine the nature of these comments or how they differ. Recent qualitative evidence suggests that maternal conversations about weight with children are diverse, and may include positive comments intended to build self-esteem and promote body acceptance in their child [27]. Examples of positive parental weight communication could include comments that their child’s health is more important than their weight, expressing reassurance rather than judgment if their child gains weight, and complimenting their child’s appearance regardless of body shape or size. Identifying what this positive parental weight-talk looks like, and how common it is, can provide a more comprehensive and nuanced understanding of parental weight communication that is currently lacking.

Another aspect of positive parental weight communication that has received almost no empirical attention is positive weight socialization, which refers to the ways that parents express their values and views about body weight or size to children, with a specific emphasis on the importance of inclusion, acceptance, and equality of people of all body sizes, and promoting awareness of weight stigma and prejudice in society. To date, parental socialization practices with respect to living with stigmatizing identities have been primarily studied in the context of family ethnic-racial socialization, defined as the intentional or unintentional ways that parents communicate their views and beliefs about race and ethnicity to children, often with the aim of instilling racial pride and helping children understand and cope with race-related issues like discrimination [28,29]. Parental racial socialization has received considerable research attention in African American families, examining the ways that parents transmit attitudes, values, and information about race and intergroup relationships [28,29]. This research illustrates that parental racial socialization can include multiple components related to cultural socialization and awareness of bias and prejudice, such as talking to children about racism, explaining instances depicting poor treatment of Blacks (e.g., in the news, viewed on TV), expressing the importance of fighting for racial equality, and taking pride in one’s racial/ethnic identity [28,29]. Several recent meta-analyses and systematic reviews of this literature conclude that parental ethnic-racial socialization is a consistent predictor of youth adjustment [30], contributing to positive mental health, behavioral, social, and academic outcomes for the youth [31,32,33].

Despite considerable evidence of weight stigma and discrimination in western societies [34,35], parental weight socialization has been neglected in the study of parent–child weight communication. While numerous studies have documented parental (primarily maternal) socialization of children in relation to dieting, body dissatisfaction, unhealthy weight preoccupation, and ideals of thinness [14,16,36,37], the literature examining how parents communicate with children about body size diversity and weight stigma is scarce [38,39,40]. Thus, it is unknown how common this parental communication practice is, how youth perceive this communication from parents, what impact it may have on their willingness to talk about weight with their parents, or how it compares to the frequency of negative or critical weight comments expressed by parents. Examining these questions can provide a more comprehensive understanding of the different types of weight communication that parents engage in with children. Furthermore, given the potential for improving youth outcomes through parental messages of body acceptance, equality, and awareness of stigma, studying the nature and prevalence of parental weight socialization messages with children is warranted.

### 1.3. Adolescent Preferences and Perceptions of Parental Weight Talk

Although the frequency of parental weight communication has been well documented, little research has examined youth preferences for parents commenting on or talking about weight. In particular, it is important to learn the extent to which youth want their parents to talk about weight with them and under what circumstances. For example, youth may feel more comfortable with these conversations if they bring it up first, if parents ask them whether it is okay to talk about it, and/or if parents are sensitive in the ways they refer to their child’s weight. On the other hand, there may be youth who never want to talk about their weight with their parents, regardless of how private or supportive the conversation is intended to be. Furthermore, these preferences may differ for girls and boys, and depending on whether comments come from mothers or fathers. Moreover, given evidence that weight-talk between parents and adolescents who are overweight is twice as high as that for parents with adolescents with a lower weight [41], preferences for weight communication may be different for youth of higher versus lower weight status or for youths engaged in weight management. Identifying these youth preferences can help inform how to best guide parents in approaching conversations about weight with their child, especially for parents who have concerns about their child’s weight and feel the need to express those concerns.

### 1.4. Contextual Aspects of Parent–Child Weight Communication

In relation to this, research is needed to obtain a more comprehensive understanding of how, and in what situations, parents and youth most commonly communicate about weight. Studies to date have not assessed different modes of communication, such as whether conversations about weight happen only face-to-face between parents and children, or whether additional modes of communication are also utilized, such as texting or posting comments on social media. While face-to-face conversations may be the default assumption, consideration of electronic modes is warranted in light of youths’ reliance on digital media [42] and texting for interacting with friends and family members. Recent evidence even suggests that digital communication (e.g., texting) may be a useful tool in promoting parent–adolescent connections [43], and that parent- and adolescent-reported handling conflicts or expressing emotional support through text messaging predicts increased feelings of relational closeness in these relationships [44]. In addition, many adolescents report feeling more comfortable expressing themselves through texting or social media than in face-to-face conversations [45]; whether this is true for emotionally charged topics like body weight has not been studied. Identifying these patterns of weight communication has important implications for the ways in which to best help parents navigate weight-related conversations with the youth.

When face-to-face conversations about weight do occur between parents and their children, we know little about what situations or settings these are most likely to be. Situations involving food or eating may elicit parent weight-talk, such as eating together at the dinner table or grocery shopping, along with situations that promote body salience, such as shopping for clothes, looking at social media, talking about sports, or going to a medical appointment. Alternatively, routine situations in family daily life, such as watching television, could also provoke parental comments about weight. Gaining a better understanding of these contextual aspects of parental weight communication, and how they may differ for parents and adolescents across variables like sex and weight status, can inform educational and intervention initiatives to improve supportive family communication about weight-related health.

### 1.5. Study Aims

In response to these identified gaps in knowledge and recent calls for research to better delineate the nature and types of parent weight communication, the present study aimed to conduct a comprehensive investigation of weight-talk in two large, racially/ethnically diverse, and independent samples of adolescents and parents. We specifically assessed parent and adolescent perspectives on how often parents engage in negative weight-talk, positive weight comments, positive weight socialization, and the most common modes of weight communication (e.g., in person, texting) and situations where weight-talk occurs. Additionally, we assessed adolescents’ preferences for parent weight communication, including how much they want their parents to talk about their weight with them and under what circumstances. We examined these key aspects of weight communication across parent source (mothers, fathers), adolescent sex (girls, boys), race/ethnicity, weight status, and whether or not adolescents were engaged in weight management. Notably, with recent calls for research to improve the representation of fathers, our sample of parents included 48% male parents, allowing for meaningful comparisons of parental sources. In line with recent and prior evidence, we hypothesized that parent weight-talk would be reported by approximately 50% of adolescents. However, given the lack of research on the positive aspects of parent weight-talk, modes and situations of parent–child weight communication, and adolescent preferences for these conversations, research questions examining these patterns across sex, race/ethnicity, weight status, and weight management were exploratory in nature and were intended to establish an initial knowledge base.

## 2. Materials and Methods

### 2.1. Participants

Two independent samples of participants were obtained for this study: a sample of parents with children aged 10–17 years old, and a sample of pre-adolescents and adolescents aged 10–17 years (unrelated to the parent sample). Participants in both samples were recruited across the United States via Qualtrics Panel Services, a company that aggregates over 20 online sample providers that have access to several million people from all 50 states. Quotas were established to achieve samples with approximately equal gender (i.e., male, female) and race/ethnicity (i.e., Black or African American, White, Latinx or Hispanic) numbers. In addition, the weight status distributions of the parent and adolescent samples were selected for rough alignment with national averages. Participants with missing/implausible BMI data, mischievous responses (e.g., bot detection, duplicate or invalid IP address) and/or if they or their children were outside the eligible age range were excluded (parent sample *n* = 184; adolescent sample *n* = 298), resulting in a final sample of 1936 parents and 2032 adolescents. 

As shown in Table 1, the final parent sample was comprised of approximately half male parents and the ethnic/racial breakdown as follows: 33% Black or African American, 32% White, 31% Latinx or Hispanic, and 4% Multiethnic or another race/ethnicity. Participants in the parent sample were, on average, 39.78 (SD = 9.71) years old, with a mean BMI of 28.29 (SD = 7.83). Demographic and anthropometric information for the adolescent sample is displayed in Table 2. Just over half (54.6%) of the adolescents were female and the ethnic/racial breakdown was as follows: 40% White, 25% Black or African American, 23% Latinx or Hispanic, and 12% Multiethnic or another race/ethnicity. Participants in this sample were, on average, 14.63 (SD = 2.46) years old, with a mean BMI percentile of 68.04 (SD = 31.05).

### 2.2. Procedure

All study procedures were approved by the Institutional Review Board at the University of Connecticut. Qualtrics Panel Services recruited the parent and adolescent samples from various sources, including social media advertisement, messages in mobile applications, member referrals, targeted email lists, and customer loyalty web portals. Survey invitations included the expected length for survey completion, as well as a description of the available incentives for participation. Interested individuals proceeded to the survey platform by clicking on the provided hyperlink. Prior to completing the survey, informed consent was obtained for all parent and adolescent participants, which included a reminder that participation was voluntary and responses would remain anonymous; parents provided consent for preadolescent participants below the age of 13. After completing a battery of survey questions pertaining to parental communication about weight with youth, lasting approximately 20–25 min, respondents were compensated for their participation with incentives managed by Qualtrics (e.g., cash, gift cards, redeemable points, vouchers). Data collection occurred from October 2021 to December 2021.

### 2.3. Measures

#### 2.3.1. Demographic Characteristics and Weight Status

Parents reported their sex, age, race/ethnicity, sexual orientation, marital status, role as caregiver (e.g., mother, father, stepmother, etc.), and highest level of education (coded as “college degree or equivalent” vs. “no college degree”). Body mass index (BMI) was calculated from parents’ self-reported height and weight, using clinical guidelines from the World Health Organization [46]. For answering survey questions in relation to their child, parents were first asked to provide their child’s sex, gender identity, date of birth, current weight and height. For parents with more than one child between the ages of 10–17 years, they were instructed to pick only one child to report on when answering the survey questions. 

Adolescent participants reported their sex, gender, sexual orientation, race/ethnicity, age (year and month they were born) and parent level of education. BMI percentile for adolescents was calculated from self-reported height and weight (and sex assigned at birth and age) based on the Centers for Disease Control and Prevention (CDC) 2000 growth charts. Finally, parents were asked whether their child is currently trying to lose weight, stay the same weight, gain weight, or not trying to do anything about his/her weight [46], followed by a yes/no question to indicate whether their child has tried to lose weight or keep from gaining weight in the past year. Adolescent participants were asked the same set of questions about their current and prior weight management.

#### 2.3.2. Frequency of Weight Communication

Frequency of parental comments about weight were measured using items previously tested in Project EAT, a population-based, 20-year longitudinal cohort study of weight-related health and associated factors in socioeconomically and ethnically diverse young people [14,47]. Parents were asked how often they or their spouse/significant other make comments to their child about his or her weight. The response options ranged from 1 = “never” to 5 = “very often”. Adolescent participants were asked how often (1) their mother and (2) their father makes comments to them about their weight, with the same 5-point response scale as above and an additional “not applicable” response if they did not live with either their mother or their father. Adolescents were also asked three questions, developed for the present study, regarding their preferences for how often, and under what circumstances, they feel comfortable talking about weight with their parent(s). Specifically, two questions asked “In general, how much do you want your mother [father] to talk about weight with you?”, with response options including “never”, “once in a while”, “sometimes”, and “often”. A third question asked, “In general, when is it okay with you for your parent(s) to talk about your weight? (select all that apply)”. Response options included “Never, I don’t want to talk about my weight with my parent(s)”, “If I bring it up first”, “If they ask me if it’s okay with me to talk about it”, “If they use words to talk about weight that I’m comfortable with”, “If they talk about my weight in a supportive way”, “If they keep it a private conversation when no one else is around”, and “Anytime—I will talk about my weight with my parents anytime”.

#### 2.3.3. Parental Weight Comments

Frequency of negative weight comments made by parents about their child’s body size or weight was measured using 12 items modified from the Fat Talk Questionnaire (FTQ) [11,48]. The original 14-item FTQ measures the frequency that women make disparaging comments about their own bodies when in the presence of female friends [48], but was adapted by Lydecker and colleagues [11] to assess how often parents comment on their own body, their child’s body, or another person’s body in the presence of their child. For the present study, we assessed parental comments made about their child’s body (not their own body or someone else’s), and eliminated two items with the potential to elicit particularly strong/negative emotional reactions (i.e., “I comment that I hate his/her whole body”; “I comment that his/her body is disgusting”). We further modified survey instructions to be applicable to parents with children of diverse body sizes in our sample; thus, rather than instructing parents to indicate how often they make comments when they notice their child “has gained weight”, parents in our sample were asked how often they make comments about their child’s body when they notice their child’s weight or body size, such as commenting “his/her arms are too flabby” or “his/her stomach is fat”. Response options ranged from 1 = “never” to 5 = “always”, and responses were averaged to provide an overall score of negative weight comments. This measure demonstrated high internal consistency with a Cronbach’s alpha of 0.98.

To assess adolescent perceptions of frequency of parental weight comments, adolescents completed the Parental Comments Questionnaire [8]. This measure asks the youth to indicate the frequency with which each of their parents have made statements to them about their weight, including both negative comments (7 items, e.g., “You need to lose weight”) and positive comments (4 items, e.g., “Your health is what is important, not your weight”). Participants completed the same set of questions twice, once for comments made by their mother, and once for comments made by their father. Response options ranged from 1 = “never” to 5 = “always”, and the Cronbach alpha values for subscales in the present sample were as follows: negative comments from mothers: α = 0.93, negative comments from fathers: α = 0.94, positive comments from mothers: α = 0.81, and positive comments from fathers: α = 0.87.

#### 2.3.4. Parental Weight Socialization

As no measures exist to assess positive weight socialization, we developed seven items for this study to examine parent communication with children specifically in the context of promoting values of body acceptance, equality and fairness of people of diverse body sizes, and awareness of weight-based prejudice. Item content was informed by prior literature on weight stigma and by measures of racial socialization, which assesses parental racial socialization messages with children, including race pride and awareness of bias [28,29]. Based on this literature, we developed our items to assess parental communication of values of equality, acceptance, diversity, and awareness of unfair prejudice and stigma. Specifically, parents were asked how often they have made various weight socialization comments to their child, including comments that people of all body sizes are equal, people of all body sizes should be treated with respect, and the importance of accepting people of all body sizes, pointing out instances of people being unfairly treated because of their body size or weight, commenting that it is unacceptable to treat people unfairly because of their body size, and talking to their child about accepting his/her own body size. Response options included “never”, “once or twice”, and “more than twice” (parent sample: α = 0.87; adolescent sample: α = 0.91). Adolescents were provided with the same questions and were asked to indicate how often their parent(s) made these types of comments to them.

#### 2.3.5. Modes and Situations of Weight Communication

To determine the most common ways and circumstances in which parental weight communication occurs, both parents and adolescents responded to a series of identical questions developed for this study. First, four questions asked parents how often they communicate with their child about his/her weight by having a conversation in-person, through text messages, using social media, and through other people (e.g., telling a doctor or other family member to talk to their child about weight). Next, parents were asked how often they talk about weight with their child in nine different daily or typical family-life situations, including eating at the dinner table, shopping for clothes, watching television, grocery shopping, talking about sports, getting ready for bed, looking at social media, when talking about healthy lifestyle behaviors, and when at the doctor’s office. Response options for all questions ranged from 1 = “never” to 5 = “always”. Adolescent participants completed the same questions, with instructions to indicate how often they talk with their parents about their weight in each of these ways and situations.

### 2.4. Analytic Plan

Analyses were conducted in SPSS, version 27. Descriptive statistics are reported for the parental weight communication variables, including the frequency and timing of weight-related comments from parents, parental weight socialization, and context of parent weight communication; results are described separately among the parent sample followed by the adolescent sample. A series of one-way analyses of variance (ANOVA) were conducted to test for demographic (i.e., sex, race/ethnicity) and weight-related (i.e., child weight status, weight management) differences in the parent weight communication variables. Male and female parents (five individuals identifying as another sex were excluded in sex difference analyses due to low prevalence), as well as male and female adolescents, were differentiated to test for sex differences in the parent and youth samples, respectively. Tests of racial/ethnic differences compared White, Black/African American, and Latinx parent- and adolescent-reports, respectively (those who indicated another race/ethnicity were excluded from the race/ethnicity difference analyses due to low prevalence). Differences in parental weight communication based on weight status compared adolescents (or parents of youth) across BMI percentile categories (<5th percentile, 5–84.9th percentile, 85–94.9th percentile, and ≥95th percentile). Finally, a dichotomous weight management variable was used to distinguish parents with a child who has engaged or not engaged in weight management in the past year, as well as adolescents who reported that they have or have not engaged in weight management in the past year. Initial analyses also considered differences as a function of a four-category child weight management variable (see “[Child] Current Weight Management Goals” in Table 1 and Table 2); however, given that a similar pattern of weight management differences were revealed when considering the four and two category variables, the four category variable is excluded for parsimony. Across all analyses, statistical significance was defined at *p* < 0.01 and listwise deletion was utilized for missing data handling.

## 3. Results

Sixty-one percent of parents (50% of mothers, 73% of fathers) reported that they or their spouse/significant other make comments to their child about his or her weight. Across racial/ethnic groups, 63% White parents, 53% Black/African American parents, and 69% Latinx parents reported making comments about their child’s weight. Sixteen percent of parents (10% of mothers, 23% of fathers; 20% of White parents, 10% of Black/African American parents, 19% of Latinx parents) indicated that they make these comments ‘often’ or ‘very often’.

Table 3 displays adolescent perceptions of, and preferences for, parental weight communication, including frequencies of these variables summarized across demographics and weight status. Overall, the majority of adolescents reported that their mother (63%) and father (54%) make comments to them about their weight (i.e., not “never”); eighteen percent and 13% indicated that these comments are made “often” or “very often” from their mother and father, respectively. For girls, 19% reported frequent (i.e., “often” or “very often”) weight comments from mothers and 12% from fathers. Among boys, 17% reported frequent weight comments from mothers and 16% from fathers. Nineteen percent of Latinx adolescents reported frequent weight comments from mothers versus 17% of White youth and 14% of Black/African American youth; frequent weight comments from fathers were reported by 18% of Latinx adolescents, 13% of White youth, and 12% of Black/African American youth. Adolescents with the highest weight status and those engaged in weight management reported the highest frequencies of weight comments from mothers and fathers.

Overall, 44% and 63% of adolescents indicated that they “never” want their mother or father, respectively, to talk about their weight with them. Adolescents who said that they never want their mother to talk about their weight reported significantly fewer negative [*t*(1880.29) = 6.76, *p* < 0.001] and fewer positive [*t*(1710.79) = 13.01, *p* < 0.001] comments from their mother. Similarly, adolescents who said they never want their father to talk about weight reported significantly fewer negative [*t*(1271.39) = 15.40, *p* < 0.001] and fewer positive [*t*(1579.77) = 14.95, *p* < 0.001] comments from their father. Among girls, 49% reported never wanting their mothers to talk about their weight and 73% reported never wanting their fathers to talk about their weight. For boys, these percentages were 36% for mothers and 49% for fathers. Similar patterns emerged across race/ethnicity and weight status, such that a higher percentage of adolescents reported never wanting their fathers to talk to them about their weight compared to their mothers. When adolescents were asked under what circumstances they feel it is okay for their parent(s) to talk about their weight, the largest proportion of this sample (44%) indicated that it is okay if they bring it up first. Approximately a third of adolescents reported that it is okay for their parent(s) to talk about their weight if they do it in a supportive way (32%) or if their parent(s) first ask them if it is okay to talk about it (31%). Approximately a quarter of adolescents noted that parent(s) talking about their weight is okay if parent(s) use words to talk about weight that they are comfortable with (26%) or if they keep it as a private conversation, when no one else is around (24%). Only 14% of adolescents reported that it is okay for their parent(s) to talk about their weight at any time. 

### 3.1. Parental Weight Comments

Table 4 displays the frequencies of parents engaging in weight comments, as reported by parents and adolescents. When considering the overall frequency of parent-reported weight comments (i.e., combined across items), differences were revealed across each of the demographic and weight-related grouping characteristics (see Table 5. For example, weight comments were reported to be more frequent by fathers (*M* = 2.36, *SD* = 1.26) compared to mothers (*M* = 1.66, *SD* = 0.98), *F*(1930) = 186.44, *p* < 0.001. In addition, parent-reported weight comments were most frequent among Latinx parents (*M* = 2.40, *SD* = 1.35), and were less common among White parents (*M* = 2.04, *SD* = 1.19), followed by Black/African American parents (*M* = 1.63, *SD* = 0.84), *F*(1853) = 69.16, *p* < 0.001. When considering their child’s weight status, parental weight comments were significantly more frequent in those with children with a BMI ≥95th percentile (*M* = 2.63, *SD* = 1.30) compared to those with children in all other weight categories, *F*(1888) = 61.41, *p* < 0.001. Parent-reported weight comments were also significantly more frequent among those with a child engaged in weight management (*M* = 2.53, *SD* = 1.25) compared to those with a child not engaged in weight management (*M* = 1.43, *SD* = 0.74), *F*(1925) = 541.72, *p* < 0.001. 

As presented in Table 4 and Table 5, overall, adolescents reported positive parental (i.e., combined across mothers and fathers) weight comments (*M* = 2.69, *SD* = 1.06) to be more frequent than negative parental weight comments (*M* = 1.91, *SD* = 1.03), *t*(1995) = 26.09, *p* < 0.001. For example, 39% of adolescents reported that their mothers (versus 31% for fathers) had frequently commented (often or very often) that their health is more important than their weight. Paired sample *t*-tests indicated that adolescents reported overall weight comments (i.e., positive and negative combined) to be more frequent from their mothers (*M* = 2.31, *SD* = 0.83) than from their fathers (*M* = 2.11, *SD* = 0.93), *t*(1752) = 14.24, *p* < 0.001. Both positive and negative comments from fathers were less frequently reported by girls than boys. In addition, boys reported more weight comments overall (i.e., positive and negative) from their mothers (*M* = 2.38, *SD* = 0.91) as compared to their fathers (*M* = 2.25, *SD* = 1.01), *t*(731) = 6.45, *p* < 0.001; similarly, girls reported more weight comments overall from their mothers (*M* = 2.26, *SD* = 0.78) as compared to their fathers (*M* = 2.00, *SD* = 0.86), *t*(1020) = 12.98, *p* < 0.001. Finally, paired sample *t*-tests revealed that boys and girls both reported positive weight comments from mothers (boys: *M* = 2.92, *SD* = 1.08; girls: *M* = 2.80, *SD* = 1.13) to be more frequent than negative comments from mothers [boys: *M* = 2.03, *SD* = 1.10, *t*(793) = −18.90, *p* < 0.001; girls: *M* = 1.93, *SD* = 1.09, *t*(1166) = −18.12, *p* < 0.001], as well as more frequent positive comments from fathers (boys: *M* = 2.61, *SD* = 1.23; girls: *M* = 2.45, *SD* = 1.23) compared to negative comments from fathers [boys: *M* = 2.04, *SD* = 1.15, *t*(743) = −12.16, *p* < 0.001; girls: *M* = 1.75, *SD* = 1.05 *t*(1035) = −15.07, *p* < 0.001].

Racial/ethnic differences emerged such that adolescent reports of negative weight comments from mothers and fathers were more frequent among Latinx adolescents compared to their White and Black/African American peers; positive weight comments from fathers were reported to be less frequent among Black/African American adolescents relative to their White and Latinx counterparts. Weight-related differences were most prevalent in regard to negative parental weight comments. For example, adolescents with a BMI ≥95th percentile, as well as those who engaged in weight management in the past year, reported significantly more frequent negative weight comments from their mothers compared to their peers with lower weight and those who had not engaged in weight management in the past year. 

### 3.2. Parental Weight Socialization

Table 6 summarizes the frequencies of parent weight socialization items, as reported by parents and adolescents. The majority of parents and adolescents indicated positive parent weight socialization at least once, with the highest prevalence within the parent sample pertaining to comments that people of all body sizes should be treated with respect, and within the adolescent sample pertaining to statements about the importance of body acceptance. The lowest prevalence across both samples pertained to pointing out examples of someone being treated in a mean or unfair way because of their body weight on social media or TV. Analyses comparing demographic and weight-related differences in parental weight socialization are presented in Table 7. Overall levels of, and group differences in, parental weight socialization (mean across items) showed no differences across demographic and weight-related variables. This consistency was documented among both the parent and adolescent samples. The only exception that emerged was for parent-reported child weight management status, such that weight socialization comments were more commonly expressed by parents with a child engaged in weight management (*M* = 2.33, *SD* = 0.47) compared to parents with a child not engaged in weight management (*M* = 2.11, *SD* = 0.66), *F*(1925) = 69.37, *p* < 0.001.

### 3.3. Context of Parent Weight Communication

Table 8 displays the frequency of parent–child weight communication across specific modes of communication (e.g., in-person, texting, social media, indirectly through another person) and within specific contexts (e.g., at the dinner table, shopping for clothes), as reported by parents and adolescents. Overall levels of, and group differences in, parental weight communication contexts (i.e., average across items) among the parent and adolescent samples are presented in Table 9. Parents and adolescents reported engaging in weight talk across different situations and modes of communication. Parents, overall, reported that in-person weight conversations with their child were most frequent (*M* = 2.88, *SD* = 1.28). Weight talk across all modes of communication were reported more frequently by fathers compared to mothers (e.g., via social media, fathers: *M* = 2.43, *SD* = 1.49; mothers: *M* = 1.69, *SD* = 1.21), and by Latinx and White parents compared to Black/African American parents (who reported all modes of communication least frequently). In-person conversations were especially frequent among parents with a child with a BMI ≥95th percentile (*M* = 3.26, *SD* = 1.19), a child who engaged in weight management in the past year (*M* = 3.44, *SD* = 1.08), and by fathers (*M* = 3.10, *SD* = 1.24). In addition, parents, on average, reported weight conversations with their child to be most frequent in situations when talking about healthy lifestyle behaviors (*M* = 3.16, *SD* = 1.15), when at the doctor’s office (*M* = 2.88, *SD* = 1.23), and when talking about sports (*M* = 2.81, *SD* = 1.30). Demographic and weight-related differences emerged across each of the communication context items; in general, across contexts, weight communication was reported to be most frequent by fathers, Latinx parents, parents with a child BMI ≥95th percentile, and among parents with a child who engaged in weight management in the past year.

Adolescents, overall, also reported that weight conversations with their parent(s) were most frequent in-person (*M* = 2.74, *SD* = 1.33), especially among adolescents who engaged in weight management in the past year (*M* = 3.03, *SD* = 1.30). Compared to girls, boys reported more frequent weight communication with parents via social media and indirectly through someone else. In addition, weight talk across all modes of communication were reported more frequently by adolescents with BMI ≥95th percentile. On average, adolescents reported weight conversations with their parent(s) to be most frequent when at the doctor’s office (*M* = 2.73, *SD* = 1.29), when shopping for clothes (*M* = 2.54, *SD* = 1.27), and when talking about healthy lifestyle behaviors (*M* = 2.52, *SD* = 1.30). Demographic differences emerged across many communication contexts, showing more frequent weight communication across several contexts for boys (compared to girls). Differences in adolescent reports of parental weight communication across contexts were most pronounced based on their weight status and weight management, with consistently higher levels of weight communication across contexts reported among adolescents with BMI ≥ 95th percentile, and among those who engaged in weight management in the past year.

## 4. Discussion

In our study, 61% of parents reported making comments to their child about his or her weight, aligning with prior evidence that weight-talk is a common parental practice [4,11], and 16% of parents reported making these comments frequently (i.e., “often” or “very often”). Similarly, adolescents in our sample reported that their mother (18%) and father (13%) make frequent comments to them about their weight. Weight comments, regardless of valence, from mothers and fathers, were reported to be more frequent among adolescent boys as compared to girls. According to both girls and boys, weight comments were more frequent from mothers compared to fathers, a pattern that was consistent across race/ethnicity and weight status. While these findings parallel prior work documenting mothers as more frequent sources of weight-talk [5,49], more than half of boys and girls in our sample reported weight comments from fathers, which is higher compared to previous studies [4,49], reiterating the importance of increased attention to father–child communication about weight. Positive weight comments from mothers and fathers were reported to be more frequent than negative weight comments among both girls and boys, suggesting that parental weight-talk is not ‘all bad’ and may in fact more often reflect positive, supportive comments. Collectively, these findings suggest that more investigation is warranted to better understand perceptions of, and responses to, weight-talk among boys, intentions of weight comments made by fathers, and the nature and impact of positive parental weight-talk.

### 4.1. Differences across Sex, Race/Ethnicity, and Body Weight

The diverse adolescent and parent samples in our study allowed for meaningful comparisons of parental weight communication across sex, race/ethnicity, and weight status, highlighting several differences across these variables. First, in contrast to previous studies documenting more frequent parental weight comments among girls compared to boys [4], we found that, compared to girls, boys reported more frequent negative and positive weight comments from fathers. Additionally, both boys and fathers reported more frequent weight-talk across different modes of communication like social media, and indirectly (through another person) than girls and mothers. Fathers also reported more frequent weight-talk with their child than mothers across all communication contexts, and in many contexts boys reported more frequent weight communication with parents than girls. Collectively, these findings highlight the salience of parental weight communication for both boys and fathers (not just girls and mothers), and the need to better understand the motivations of fathers for engaging in weight-talk. Some evidence suggests that fathers are more likely than mothers to comment on the specific body parts of their child [49], though some of the reasons for these comments have not been well-studied in fathers. For example, recent qualitative evidence suggests that mothers of children with a higher body weight feel guilt and associated stigma for their child’s body size [50], which could contribute to their increased engagement in weight-talk compared to mothers of youth with lower body weights. Examining these experiences and other motivations for weight talk among fathers is an important direction for future research.

Secondly, several differences have emerged in patterns of weight communication across race/ethnicity of parents and adolescents. Engaging in parental weight comments was most frequently reported by Latinx parents, followed by White then Black/African American parents. Similarly, Latinx adolescents reported more frequent negative weight comments from parents than their White and Black/African American peers. While no differences across race/ethnicity of adolescents emerged for the frequency of positive weight comments from mothers, Black/African American adolescents reported significantly less frequent positive weight comments from fathers than White or Latinx peers. Thus, while the presence of weight communication was commonly reported across these groups, parent and adolescent reports of the frequency of parental weight-talk was generally lower for Black/African American parents and was highest for Latinx parents. These patterns also persisted for contextual aspects of weight communication, with Latinx parents and adolescents reporting more frequent weight conversations across several communication modes and most contexts compared to parents and adolescents who identified as White or Black/African American. 

The reason for more frequent weight-talk among Latinx parents in our study is unclear, but is similar to prior research documenting weight conversations as more common among Latinx parents than other race/ethnicities [5] and more frequent hurtful weight-related comments from family members reported by Hispanic young adults than those from other racial/ethnic backgrounds [25]. Levels of acculturation could be informative to examine in future work, as one study found that more acculturated Mexican mothers expressed greater concern for their child’s weight than less acculturated mothers [51], which could affect their engagement in weight-talk. Alternatively, differences in cultural awareness of consequences of weight-talk and/or views that weight talk is a way to show affection could play a role. It is also unclear why weight-talk was least frequent among Black/African American parents. Some qualitative evidence found that 50% of Black/African American parents reported avoiding weight-talk because of their own past experience of weight teasing and not wanting to repeat that experience for their child, or because they wanted their children to have good manners and respect for other people [52]. Other mixed-methods evidence points to engagement in weight-talk among Black/African American parents as a way of addressing concerns about their child’s health [49]. With relatively little research to date examining the nature of parental weight communication in racial/ethnic minorities families, our findings highlight the need for more research to understand the meanings, motivations, and values that parents of different racial/ethnic backgrounds associate with weight-talk.

Third, for adolescents at a high body weight, and/or who are trying to lose weight or prevent gaining weight, parental weight comments were particularly salient and frequent in our samples. Parent-reported frequency of negative comments about their child’s weight were highest among parents of a child with BMI ≥ 95th percentile or engaged in weight management. Similarly, adolescents with a BMI ≥ 95th percentile or engaged in weight management reported the highest frequency of negative comments about their weight made by both mothers and fathers. This parallels prior evidence showing increased odds of parents talking about their child’s weight with youth who have a higher weight status [5,12,49] and potentially different types of comments that parents engage in depending on whether they have a child with or without overweight/obesity [13]. Given the increased vulnerability to weight-based stigma and victimization among youth with a higher BMI [53], our findings underscore the importance of reducing negative parental weight-talk in families with youth who have obesity and/or who are trying to manage their weight, and targeting parent communication in family-based weight-loss interventions. Interestingly, there were no differences in the frequency of parental positive weight comments or weight socialization reported by adolescents who were engaged in weight management against those who were not. However, parent reports indicated a higher frequency of weight socialization comments if their child was engaged in weight management compared to those who were not. Thus, at least from parent perspectives, there may be some awareness of weight stigma and the importance of communicating body acceptance and/or diversity to their child when he or she is actively trying to manage his/her weight. It will be important for future research to identify how positive and negative comments impact youth at different body sizes and whether these comments differentially affect weight management outcomes for the youth. For example, some research has found that adolescents with obesity in a behavioral weight control intervention lost more weight when negative maternal comments about weight or appearance were limited [54]. Whether the presence of positive weight comments affects these outcomes in adolescents and/or lessens the adverse impact of negative comments is unknown.

### 4.2. Positive Weight Communication

Our study provides novel insights on the frequency and nature of positive weight communication by parents. Among both adolescent boys and girls in our sample, positive weight comments from parents were reported to be more frequent than negative parental comments about weight, echoing recent evidence [4]. Furthermore, while positive comments were reported to be more frequent from mothers than from fathers, a high percentage of adolescents (19–39%) reported various positive comments from fathers at least “often”, or “very often”. Particularly noteworthy is that high percentages of adolescents reported that their fathers (31%) and mothers (39%) had commented (at least often) that their health is more important than their weight. Encouraging healthy behaviors without referencing weight control has been increasingly recommended in the literature and public health messaging [55,56], and aligns with evidence that health-focused conversations are associated with more healthful behaviors, positive outcomes, and are less harmful for adolescents than weight-focused talk [13,17].

The majority of both parents and adolescents reported that parents had expressed positive weight socialization comments at least once. The most frequent type of weight socialization reported by parents was that people of all body sizes should be treated with respect, and youth reported statements pertaining to the importance of body acceptance to be most frequent. Unlike other weight communication variables analyzed in this study, there were no differences in parent or adolescent sex, race/ethnicity, or weight status in frequency of parental weight socialization, suggesting that many parents are communicating messages about body diversity and acceptance. Collectively, these findings are encouraging, and indicate that while most parents may be engaging in weight-talk, this communication is not necessarily always negative or ‘all bad.’ Most research to date has focused on negative and critical aspects of parental weight-talk, and our findings suggest the importance of further examining the prevalence and nature of positive weight communication and its potential impact on youth. In particular, it will be important to determine whether, and in what ways, positive comments and weight socialization contribute to more positive outcomes and adjustments in youth. 

Despite these cautiously optimistic findings, many adolescents in our sample reported that they ‘never’ want their mother (44%) or father (63%) to talk to them about their weight. As these preferences for no parental weight talk are higher than the percent of adolescents, indicating that they, in general, ‘never’ assent to their parents talking about their weight (21%), it is important that additional research examines adolescents’ reasons and emotions related to not wanting to talk about weight with their parents. Across adolescent sex, race/ethnicity, and weight status, the trend of never wanting parents to talk about weight was particularly the case for fathers. This suggests that there may be distinct aspects of weight-talk from fathers that induce more distress or discomfort among adolescents than weight-focused conversations with mothers. As we did not ask adolescents to explain their responses, it will be important for future work to identify reasons why adolescents desire a heightened avoidance of weight communication with fathers, and whether fathers are engaging in specific aspects of weight-talk (e.g., body criticism or teasing) that are particularly distressing to adolescents. More broadly, these findings indicate that even if parents express positive weight comments more frequently than negative comments, the positive messages may not outweigh the impact of the negative ones, contributing to youth wanting to avoid weight conversations altogether. Still, our findings offer insights into circumstances that adolescents may feel more comfortable talking with parents about weight, primarily when they can bring up the topic themselves (e.g., not parent initiated), if parents ask them first if it is okay to talk and talk in a supportive way, and use words to describe their weight that they feel comfortable with.

### 4.3. Contextual Components of Weight Communication

Finally, the study findings offer new insights into other contextual aspects of parental weight communication. Both parents and adolescents in our study reported in-person conversations to be the most common mode of communicating about weight, but also reported engaging in weight-talk via texting, social media, and indirectly through another person (e.g., family member). Similarly, weight communication was reported by parents and adolescents across a range of different daily life contexts, with demographic and weight differences emerging. While some prior evidence has highlighted family meals as the most frequent context for weight conversations [57], our study identified other family situations in which weight-talk was more common. For example, weight communication was particularly salient, while clothes shopping among adolescents with high BMI; when talking about sports for boys; and grocery shopping among adolescents engaged in weight management. These findings highlight the many daily contexts in which parent weight communication occurs, which may be more or less salient depending on sex, race/ethnicity, and weight status. Targeting these contextual components of weight-talk may help tailor interventions for different families to promote more supportive parental communication through different formats (e.g., texting) and across different daily situations. It will also be informative for future research to examine additional situational contexts where family weight communication could be salient, such as wearing bathing suits at the beach or dressing up for special events.

### 4.4. Strengths, Limitations, and Future Directions

Our study has a number strengths, including the use of adolescent and parent samples with both males and females, individuals of diverse racial/ethnic backgrounds and different weight categories. In addition, our study provides a comprehensive examination of both positive and negative parental weight comments, including the nature and frequency of these comments. Furthermore, key variables examined in our study directly respond to calls for research to include a stronger representation of fathers, to identify adolescent preferences for weight communication with parents, and to examine contextual aspects of parental weight communication with the youth. Collectively, our findings offer important insights into the nature and frequency of different types of parental weight communication across key demographic characteristics and relevant weight variables. Limitations include the use of two independent samples of parents and adolescents rather than parent–child dyads, as well as a reliance on cross-sectional, self-report data. Additionally, we did not examine gender or sexual identity of adolescents or include younger children in our sample, for whom perspectives of weight communication may differ from the adolescents in our sample.

While our study provides a comprehensive investigation of the nature of parent–adolescent weight communication, there are several future directions for research that can further expand this area of study. First, we did not assess physical activity, and it is possible that parent and adolescent levels of, or engagement in, physical activity, could contribute to the frequency and nature of parental weight communication. For example, some evidence suggests that parents play a stronger role in supporting boys to be more physically active than girls [58], and prior studies have found fathers to be more involved in their son’s (rather than daughter’s) physical activity [59,60]. These findings could help explain why some adolescents in our study aim to avoid talking about their weight with fathers, and highlights physical activity as an important topic of study for future research examining weight communication in families. Second, it was beyond the scope of our analyses to examine other parental characteristics that may increase or decrease their engagement in weight communication. For example, body dissatisfaction may be elevated for people with a higher weight [61], and could affect the frequency and nature of parental weight communication. Additionally, parenting styles and more general communication patterns could influence the ways that parents talk with their children about weight. It will be informative to examine the roles of body dissatisfaction and other parental characteristics in contributing to the nature of parental weight comments and their motivations for engaging in or avoiding weight communication. Third, it will be important for future studies to more thoroughly examine the nature and contexts of both health-focused and weight-focused parental communication, and whether adolescents are more amenable to talking to parents about their health rather than their weight. Parents may engage in both of these types of communication [13], and given the strong links between parent and child health-related fitness [62], improving our understanding of the ways in which parental comments about health versus weight intersect and affect adolescents’ wellbeing is a key priority for future work. Finally, in light of evidence that adolescents face weight stigma from other adults besides parents, such as teachers or coaches [63], future research is needed to examine the nature, frequency, and impact of weight communication from educators and coaches directed at the youth.

## 5. Conclusions

The findings of our study indicate that positive weight comments from parents are more frequent than negative comments, although both are commonly reported by parents and adolescents across sex, race/ethnicity, and weight status. More frequent weight-talk reported among Latinx parents and adolescents, and by fathers and among boys, reiterates the need for more studies to examine the nature and patterns of weight communication in males and in families with diverse racial/ethnic backgrounds. As parental weight-talk was particularly frequent (and negative) for adolescents with a high weight and/or those managing their weight, family-based weight management programs should prioritize efforts to help parents engage in more supportive communication. In our samples, weight conversations were reported across multiple modes of communication and situational contexts, highlighting the importance of attending to the nuanced ways in which weight-talk surfaces in parent–adolescent relationships. While our study suggests that parents are communicating positive messages of respect for people of diverse body sizes, many adolescents remain wary of any parental communication about their weight. Adolescent input on factors to improve their comfort level in these conversations, such as allowing them to initiate conversations and ensuring that parents know how to engage in supportive ways using sensitive language, can inform guidance and resources for parents to promote more positive dialogue. More broadly, the results of our study have implications for healthcare providers and educators who have opportunities to educate parents about weight-talk. Helping parents distinguish between positive and negative weight comments, identify which situations they tend to make these comments, and understand perspectives of adolescents in response to their weight-talk, may provide initial and concrete steps towards more supportive parent communication.

## Figures and Tables

**Table 1:** Demographic and anthropometric information for the parent sample (*n* = 1936).

Variable	M	SD
Age	39.78	9.71
BMI	28.29	7.83
	*n*	*%*
Sex		
Male	932	48.1
Female	999	51.6
Other	5	0.3
Race/Ethnicity		
White, non-Hispanic, non-Latino	618	31.9
Black or African American	630	32.5
American Indian or Alaska Native	12	0.6
Asian or Pacific Islander	18	0.9
Latino, Hispanic, or Mexican-American	606	31.3
Multiethnic	38	2.0
Other	14	0.7
Sexual Orientation ^a^		
Heterosexual or Straight	1749	90.5
Gay, Lesbian, or Homosexual	57	3.0
Bisexual	110	5.7
Other	16	0.8
Marital Status ^b^		
Married	1121	57.9
Divorced	174	9.0
Widowed	53	2.7
Separated	63	3.3
Never Married	524	27.1
Level of Education		
College Degree (or Above)	879	45.4
No College Degree	1057	54.6
BMI Category		
<18.5 kg/m^2^	124	6.4
18.5–24.9 kg/m^2^	603	31.1
25–29.9 kg/m^2^	582	30.1
≥30 kg/m^2^	627	32.4
Child Current Weight Management Goals ^c^		
Lose Weight	388	20.1
Stay the Same Weight	662	34.2
Gain Weight	247	12.8
Not Trying to Do Anything About Weight	638	33.0
Child Weight Management in Past Year ^d^		
Yes	1004	52.1
No	922	47.9

Note. ^a^ 4 missing. ^b^ 1 missing. ^c^ 1 missing. ^d^ 10 missing. Due to rounding, percentages do not always add up to 100.

**Table 2:** Demographic and anthropometric information for the adolescent sample (*n* = 2032).

Variable	M	SD
Age	14.63	2.46
BMI percentile	68.04	31.05
	*n*	*%*
Sex		
Male	825	40.6
Female	1207	59.4
Gender		
Male	816	40.2
Female	1109	54.6
Transgender	34	1.7
Do not identify myself as female, male, or transgender	73	3.6
Race/Ethnicity		
White, non-Hispanic, non-Latino	816	40.2
Black or African American	502	24.7
American Indian or Alaska Native	15	0.7
Asian or Pacific Islander	87	4.3
Latino, Hispanic, or Mexican-American	476	23.4
Multiethnic	102	5.0
Other	34	1.7
Sexual Orientation ^a^		
Heterosexual or Straight	1546	76.2
Gay, Lesbian, or Homosexual	89	4.4
Bisexual	246	12.1
Other	63	3.1
Not sure, questioning	86	4.2
Parental Level of Education ^b^		
College Degree (or Above)	689	34.9
No College Degree	1288	65.1
BMI Category		
BMI < 5th%ile	106	5.2
BMI 5–84.9th%ile	1056	52.0
BMI 85–94.9th%ile	360	17.7
BMI ≥ 95th%ile	510	25.1
Current Weight Management Goals ^c^		
Lose Weight	812	40.0
Stay the Same Weight	475	23.4
Gain Weight	222	10.9
Not Trying to Do Anything About Weight	522	25.7
Weight Management in Past Year ^d^		
Yes	1080	53.5
No	937	46.5

Note. ^a^ 2 missing. ^b^ 55 missing. ^c^ 1 missing. ^d^ 15 missing. Due to rounding, percentages do not always add up to 100.

**Table 3:** Adolescent perceptions of and preferences for parental weight communication.

		Sex		Race/Ethnicity			Weight Status				Weight Management	
	Overall	Boys	Girls	White	Black or African American	Latinx	BMI < 5th %ile	BMI 5–84.9th %ile	BMI 85–94.9th %ile	BMI ≥ 95th %ile	No	Yes
	%	%	%	%	%	%	%	%	%	%	%	%
How often does your mother make comments to you about your weight? ^a^												
Never	37	36	37	39	39	31	33	43	37	24	51	24
Rarely	22	22	21	22	20	24	20	21	23	23	22	21
Sometimes	24	25	23	21	27	27	26	22	25	27	18	29
Often	10	11	10	11	7	11	15	9	9	14	5	15
Very often	8	6	9	6	7	8	6	6	7	13	4	11
How often does your father make comments to you about your weight? ^b^												
Never	46	42	49	48	51	38	34	54	46	33	61	33
Rarely	20	20	21	18	20	24	25	19	24	20	18	22
Sometimes	20	23	18	21	17	21	20	18	19	25	14	25
Often	7	9	6	7	6	9	17	6	6	11	3	11
Very often	6	7	6	6	6	9	4	4	6	12	3	9
In general, how much do you want your mother to talk about your weight with you?												
Never	44	36	49	45	45	38	43	47	39	40	52	37
Once in a While	36	40	33	33	37	38	37	35	38	35	35	37
Sometimes	15	19	13	15	15	19	14	13	18	19	12	18
Often	5	5	5	7	3	4	7	4	5	6	2	8
In general, how much do you want your father to talk about your weight with you?												
Never	63	49	73	64	68	53	62	66	59	61	71	57
Once in a While	22	29	18	21	21	26	21	22	24	22	20	25
Sometimes	11	18	7	10	9	18	11	10	14	13	8	14
Often	3	5	2	5	2	3	6	3	3	5	1	5
In general, when it is okay with you for your parent(s) to talk about your weight? [select all that apply]												
Never.	21	15	25	20	24	19	21	22	19	21	23	20
If I bring it up first.	44	42	45	43	43	46	43	44	47	40	42	45
If they ask me if it’s okay with me to talk about it.	31	33	30	31	28	34	34	30	30	35	28	34
If they use words to talk about weight that I am comfortable with.	26	27	26	26	22	31	30	24	28	29	20	32
If they talk about my weight ina supportive way.	32	33	31	33	27	35	27	29	34	38	27	37
If they keep it a private conversation, when no one else is around.	24	21	26	26	17	25	23	22	27	27	19	29
Anytime.	14	17	12	15	16	11	16	16	14	9	19	10

Note. ^a^ Excludes adolescents who reported that they do not live with their mother. ^b^ Excludes adolescents who reported that they do not live with their father. Due to rounding, percentages do not always add up to 100.

**Table 4:** Frequency and types of parental weight comments.

	Never	Rarely	Sometimes	Often	Always
	%	%	%	%	%
Parent report					
In general, when I notice my child’s weight or body size:					
I comment that his/her arms are too flabby.	63.3	8.2	11.3	11.9	5.4
2. I comment that his/her stomach is fat.	59.1	10.4	12.7	11.7	6.2
3. I comment on his/her body compared to thin models in magazines.	65.8	7.4	10.5	11.2	5.1
4. I comment that his/her body is out of proportion.	63.9	9.1	10.3	10.3	6.3
5. I comment that he/she is fat.	63.6	8.1	9.7	12.1	6.6
6. I comment that he/she should not be eating fattening foods.	45.0	12.2	18.4	14.4	9.9
7. I comment that he/she has gained weight.	46.5	15.3	18.6	13.6	6.1
8. I comment that his/her clothes are too tight.	47.5	13.7	19.7	12.8	6.4
9. I comment that he/she needs to stop eating so much.	50.2	13.9	16.2	13.1	6.6
10. I comment on his/her body compared to his/her friends’ bodies.	62.7	9.2	10.3	12.1	5.7
11. I comment that he/she must feel pressure to be thin.	63.0	8.4	11.1	11.1	6.3
12. I comment that he/she is not in shape.	56.3	11.4	13.0	11.2	8.1
Adolescent report					
How often has your mother said: ^a^	Never	Rarely	Sometimes	Often	Always
[Negative comments]	%	%	%	%	%
“You need to lose weight”	53.9	12.7	15.2	11.0	7.2
2. “You look great, but you could look even better if you lost some weight”	59.7	10.4	14.0	9.3	6.7
3. “You look like you’ve put on weight, you should watch what you eat”	55.7	14.0	13.6	9.8	6.9
4. “If you want to look good you need to work out more”	58.0	11.0	13.5	10.1	7.5
5. “If you eat that you’ll get fat”	58.7	11.1	13.2	10.0	7.0
6. “Your figure is not important to me, but you would feel better if you lost some weight”	61.4	13.4	12.1	8.2	4.9
7. “You look great, you must’ve lost weight!”	52.6	12.2	19.5	10.5	5.3
[Positive comments]					
8. “It’s ok if you put on some weight, don’t even worry about it”	41.2	15.1	26.4	11.3	6.0
9. “You always look wonderful”	17.4	9.0	20.9	25.4	27.4
10. “You need to make sure you eat enough while you’re still growing”	28.2	11.9	25.7	19.2	15.1
11. “Your health is what is important not your weight”	26.4	11.5	22.6	18.2	21.2
How often has your father said: ^b^	Never	Rarely	Sometimes	Often	Always
[Negative comments]	%	%	%	%	%
“You need to lose weight”	59.8	9.4	14.3	10.8	5.7
2. “You look great, but you could look even better if you lost some weight”	64.0	9.0	11.7	9.3	6.0
3. “You look like you’ve put on weight, you should watch what you eat”	62.3	9.6	13.3	9.2	5.5
4. “If you want to look good you need to work out more”	59.9	10.1	14.9	9.7	5.4
5. “If you eat that you’ll get fat”	62.7	8.7	13.0	8.8	6.8
6. “Your figure is not important to me, but you would feel better if you lost some weight”	65.6	9.2	12.4	8.1	4.6
7. “You look great, you must’ve lost weight!”	60.9	9.9	14.3	9.5	5.4
[Positive comments]					
8. “It’s ok if you put on some weight, don’t even worry about it”	50.1	13.2	17.9	11.7	7.1
9. “You always look wonderful”	31.9	9.9	19.5	18.8	19.9
10. “You need to make sure you eat enough while you’re still growing”	39.0	11.4	21.5	14.8	13.3
11. “Your health is what is important not your weight”	39.6	10.7	18.3	15.6	15.8

^a^ Excludes youth who reported that they do not live with their mother. ^b^ Excludes youth who reported that they do not live with their father. Due to rounding, percentages do not always add up to 100.

**Table 5:** Demographic and weight-related differences in frequency of parental weight comments.

		Sex Differences		Race/Ethnicity Differences			(Child) Weight Status Differences				(Child) Weight Management Differences	
	Overall *M* *(SD)*	Fathers / Boys *M* *(SD)*	Mothers / Girls *M* *(SD)*	White *M* *(SD)*	Black or African American *M* *(SD)*	Latinx *M* *(SD)*	BMI < 5th %ile *M* *(SD)*	BMI 5–84.9th %ile *M* *(SD)*	BMI 85–94.9th %ile *M* *(SD)*	BMI ≥ 95th %ile *M* *(SD)*	No *M* *(SD)*	Yes *M* *(SD)*
Parent report	2.00 (1.18)	2.36 ^a^ (1.26)	1.66 ^b^ (0.98)	2.04 ^a^ (1.19)	1.63 ^b^ (0.84)	2.40 ^c^ (1.35)	1.97 ^a^ (1.16)	1.75 ^a^ (1.03)	1.92 ^a^ (1.10)	2.63 ^b^ (1.30)	1.43 ^a^ (0.74)	2.53 ^b^ (1.25)
Adolescent report												
Negative—Mom	1.97 (1.10)	2.03 ^a^ (1.10)	1.93 ^a^ (1.09)	1.92 ^a^ (1.11)	1.89 ^a^ (1.03)	2.16 ^b^ (1.13)	1.93 ^a^ (1.14)	1.79 ^a^ (1.03)	1.95 ^a^ (1.03)	2.36 ^b^ (1.17)	1.52 ^a^ (.82)	2.35 ^b^ (1.16)
Negative—Dad	1.87 (1.10)	2.04 ^a^ (1.15)	1.75 ^b^ (1.05)	1.82 ^a^ (1.09)	1.79 ^a^ (1.02)	2.10 ^b^ (1.17)	1.91 ^abc^ (1.18)	1.70 ^a^ (1.02)	1.89 ^b^ (1.06)	2.21 ^c^ (1.19)	1.48 ^a^ (0.83)	2.19 ^b^ (1.19)
Positive—Mom	2.85 (1.11)	2.92 ^a^ (1.08)	2.80 ^a^ (1.13)	2.89 ^a^ (1.09)	2.77 ^a^ (1.11)	2.91 ^a^ (1.11)	2.99 ^a^ (1.06)	2.88 ^a^ (1.11)	2.87 ^a^ (1.07)	2.75 ^a^ (1.15)	2.85 ^a^ (1.12)	2.84 ^a^ (1.10)
Positive—Dad	2.52 (1.24)	2.61 ^a^ (1.23)	2.45 ^b^ (1.23)	2.57 ^a^ (1.25)	2.36 ^b^ (1.18)	2.64 ^a^ (1.24)	2.84 ^a^ (1.34)	2.55 ^ab^ (1.23)	2.49 ^ab^ (1.19)	2.40 ^b^ (1.24)	2.46 ^a^ (1.24)	2.56 ^a^ (1.23)

Note. Values within the same row and subgrouping not sharing the same letter are significantly different from each other at *p* < 0.01.

**Table 6:** Frequency of parental weight socialization.

	Never	Once or Twice	More than Twice
	%	%	%
Parent report			
How often have you:			
Said to your child that people of all body sizes are equal.	28.3	34.8	36.9
2. Told your child that people of all body sizes should be treated with respect.	16.6	26.9	56.5
3. Talked to your child about the importance of accepting people no matter what their body size is.	16.8	30.1	53.0
4. Told your child that people are sometimes treated unfairly because of their body size.	19.1	31.6	49.4
5. Pointed out to your child an example of someone being treated in a mean or unfair way because of their body weight on social media or TV.	31.3	40.7	28.0
6. Commented to your child that it’s not ok to treat people unfairly because of their body size.	21.4	32.3	46.3
7. Talked to your child about accepting his/her body size.	21.7	32.6	45.8
Adolescent report			
Please indicate how frequently your parent(s) have:			
Said that people of all body sizes are equal.	38.2	32.1	29.7
2. Said that people of all body sizes should be treated with respect.	27.6	29.6	42.8
3. Talked about the importance of accepting people no matter what their body size is.	29.1	27.1	43.8
4. Said that people are sometimes treated unfairly because of their body size.	29.0	32.2	38.8
5. Pointed out an example of someone being treated in a mean or unfair way because of their body weight on social media or TV.	39.4	32.8	27.8
6. Commented that it’s not ok to treat people unfairly because of their body size.	30.5	31.8	37.7
7. Said that it’s important to accept your body.	26.7	30.0	43.3

Note. Due to rounding, percentages do not always add up to 100.

**Table 7:** Demographic and weight-related differences in parental weight socialization.

Parent Report	Mean (SD)	F	p	Adolescent Report	Mean (SD)	F	p
Overall	2.23 (0.58)			Overall	2.06 (0.66)		
Sex		2.64	0.104	Sex		2.81	0.094
Male	2.20 (0.54)			Boys	2.09 (0.64)		
Female	2.25 (0.62)			Girls	2.04 (0.67)		
Race/Ethnicity		1.46	0.234	Race/Ethnicity		1.99	0.137
White	2.21 (0.57)			White	2.10 (0.65)		
Black or African American	2.22 (0.63)			Black or African American	2.03 (0.67)		
Latinx	2.27 (0.53)			Latinx	2.10 (0.64)		
(Child) Weight Status		1.38	0.247	Weight Status		0.59	0.620
BMI < 5th %ile	2.19 (0.61)			BMI < 5th %ile	2.04 (0.64)		
BMI 5–84.9th %ile	2.21 (0.61)			BMI 5–84.9th %ile	2.07 (0.67)		
BMI 85–94.9th %ile	2.25 (0.55)			BMI 85–94.9th %ile	2.10 (0.66)		
BMI ≥ 95th %ile	2.27 (0.54)			BMI ≥ 95th %ile	2.04 (0.64)		
(Child) Weight Management Past Year		69.37	<0.001	Weight Management Past Year		1.39	0.239
Yes	2.33 (0.47)			Yes	2.05 (0.65)		
No	2.11 (0.66)			No	2.08 (0.68)		

**Table 8:** Parent and adolescent reports of contextual aspects of parental weight communication.

	Never	Rarely	Sometimes	Often	Very Often
	%	%	%	%	%
Parent report					
How often do you communicate with your child about his/her weight:					
By talking in person with him/her.	19.5	19.1	26.8	23.1	11.5
2. Through text messages.	51.5	12.6	15.3	12.3	8.3
3. Using social media (e.g., Instagram).	58.4	8.4	11.3	14.2	7.7
4. Through other people (e.g., I tell a doctor or other family member to talk to them about it).	46.9	13.1	18.2	13.8	8.0
How often do you talk about body weight with your child:					
5. At the dinner table.	34.7	20.2	21.6	17.0	6.5
6. When shopping for clothes.	22.1	21.1	28.1	19.0	9.7
7. When watching television.	33.9	22.5	21.0	15.6	7.0
8. When grocery shopping.	30.4	19.5	23.7	17.5	8.8
9. When talking about healthy lifestyle behaviors.	9.7	16.6	35.2	25.3	13.2
10. When at the doctor’s office.	18.0	18.7	31.3	21.7	10.3
11. When talking about sports.	22.5	17.6	27.3	21.9	10.7
12. When looking at social media.	44.7	15.1	16.5	15.1	8.6
13. When getting ready for bed.	45.5	13.6	15.3	16.1	9.6
	Never	Rarely	Sometimes	Often	Very Often
Adolescent report	%	%	%	%	%
How often do you communicate about your weight with your parent(s):					
By having a conversation in person.	25.2	16.9	28.2	18.1	11.7
2. Through text messages.	58.5	15.1	13.9	8.4	4.1
3. Using social media (e.g., Instagram)	70.3	10.3	9.6	6.7	3.1
4. Through other people (e.g., I tell a doctor or other family member to talk to them about it).	67.4	11.0	11.6	5.9	4.1
How often do you talk about your weight with your parent(s):					
5. At the dinner table.	54.1	17.2	16.4	9.0	3.3
6. When shopping for clothes.	29.5	17.1	30.7	14.9	7.8
7. When watching television.	57.8	15.3	15.0	8.6	3.3
8. When grocery shopping.	50.8	17.1	18.4	9.1	4.5
9. When talking about healthy lifestyle behaviors.	32.4	15.7	28.1	15.9	8.0
10. When at the doctor’s office.	24.5	15.6	32.2	17.4	10.3
11. When talking about sports.	49.2	13.0	20.4	11.7	5.8
12. When looking at social media.	58.3	13.0	14.2	8.9	5.5
13. When getting ready for bed.	64.1	13.3	12.4	6.2	3.9

Note. Due to rounding, percentages do not always add up to 100.

**Table 9:** Demographic and weight-related differences in frequency of parental weight communication across modes and contexts.

		Sex Differences		Race/Ethnicity Differences			(Child) Weight Status Differences				(Child) Weight Management Differences	
	Overall M (SD)	Fathers / Boys M (SD)	Mothers / Girls M (SD)	White M (SD)	Black or African American M (SD)	Latinx M (SD)	BMI < 5th %ile M (SD)	BMI 5–84.9th %ile M (SD)	BMI 85–94.9th %ile M (SD)	BMI ≥ 95th %ile M (SD)	No M (SD)	Yes M (SD)
**Parent report**												
Modes of Communication												
In-person conversation	2.88 (1.28)	3.10 ^a^ (1.24)	2.68 ^b^ (1.29)	2.95 ^a^ (1.30)	2.67 ^b^ (1.24)	3.09 ^a^ (1.26)	2.81 ^ab^ (1.31)	2.72 ^a^ (1.30)	2.94 ^b^ (1.23)	3.26 ^c^ (1.19)	2.26 ^a^ (1.21)	3.44 ^b^ (1.08)
Text messages	2.13 (1.38)	2.49 ^a^ (1.46)	1.79 ^b^ (1.20)	2.27 ^a^ (1.45)	1.74 ^b^ (1.11)	2.46 ^a^ (1.47)	2.18 ^a^ (1.43)	1.94 ^a^ (1.28)	2.01 ^a^ (1.29)	2.65 ^b^ (1.49)	1.56 ^a^ (1.01)	2.66 ^b^ (1.46)
Social media	2.04 (1.40)	2.43 ^a^ (1.49)	1.69 ^b^ (1.21)	2.21 ^a^ (1.46)	1.61 ^b^ (1.11)	2.38 ^a^ (1.51)	2.09 ^a^ (1.44)	1.85 ^a^ (1.30)	1.95 ^a^ (1.34)	2.50 ^b^ (1.51)	1.47 ^a^ (.96)	2.56 ^b^ (1.52)
Indirectly	2.23 (1.37)	2.59 ^a^ (1.43)	1.89 ^b^ (1.21)	2.33 ^a^ (1.39)	1.83 ^b^ (1.14)	2.59 ^c^ (1.47)	2.21 ^a^ (1.40)	2.00 ^a^ (1.26)	2.13 ^a^ (1.32)	2.80 ^b^ (1.44)	1.61 ^a^ (1.02)	2.78 ^b^ (1.41)
Context of Communication												
Dinner table	2.40 (1.29)	2.75 ^a^ (1.28)	2.05 ^b^ (1.20)	2.45 ^a^ (1.23)	2.06 ^b^ (1.13)	2.75 ^c^ (1.41)	2.46 ^a^ (1.31)	2.20 ^a^ (1.23)	2.25 ^a^ (1.23)	2.84 ^b^ (1.32)	1.89 ^a^ (1.07)	2.73 ^b^ (1.31)
Clothes shopping	2.73 (1.27)	3.02 ^a^ (1.28)	2.44 ^b^ (1.18)	2.75 ^a^ (1.27)	2.54 ^b^ (1.16)	2.93 ^a^ (1.33)	2.60 ^a^ (1.18)	2.52 ^a^ (1.20)	2.59 ^a^ (1.24)	3.27 ^b^ (1.27)	2.20 ^a^ (1.07)	3.07 ^b^ (1.27)
Watching TV	2.39 (1.28)	2.70 ^a^ (1.28)	2.08 ^b^ (1.21)	2.45 ^a^ (1.27)	2.03 ^b^ (1.12)	2.71 ^c^ (1.37)	2.42 ^a^ (1.30)	2.16 ^a^ (1.20)	2.33 ^a^ (1.23)	2.87 ^b^ (1.35)	1.82 ^a^ (.99)	2.75 ^b^ (1.31)
Grocery shopping	2.55 (1.32)	2.84 ^a^ (1.31)	2.25 ^b^ (1.26)	2.52 ^a^ (1.26)	2.26 ^b^ (1.21)	2.90 ^c^ (1.41)	2.54 ^a^ (1.37)	2.27 ^a^ (1.22)	2.50 ^a^ (1.24)	3.10 ^b^ (1.35)	1.90 ^a^ (1.04)	2.95 ^b^ (1.32)
Talking about healthy lifestyle	3.16 (1.15)	3.33 ^a^ (1.12)	2.99 ^b^ (1.14)	3.26 ^a^ (1.13)	2.91 ^b^ (1.13)	3.32 ^a^ (1.14)	3.08 ^a^ (1.17)	3.05 ^a^ (1.14)	3.12 ^a^ (1.09)	3.45 ^b^ (1.11)	2.65 ^a^ (1.06)	3.47 ^b^ (1.08)
Doctor’s office	2.88 (1.23)	3.07 ^a^ (1.23)	2.68 ^b^ (1.21)	2.88 ^a^ (1.26)	2.63 ^b^ (1.19)	3.13 ^c^ (1.20)	2.83 ^a^ (1.25)	2.67 ^a^ (1.21)	2.82 ^a^ (1.15)	3.32 ^b^ (1.20)	2.36 ^a^ (1.12)	3.20 ^b^ (1.19)
^ ^ Talking about sports	2.81 (1.30)	3.13 ^a^ (1.26)	2.47 ^b^ (1.25)	2.83 ^a^ (1.28)	2.56 ^b^ (1.22)	3.05 ^c^ (1.34)	2.69 ^a^ (1.25)	2.62 ^a^ (1.26)	2.72 ^a^ (1.30)	3.25 ^b^ (1.28)	2.28 ^a^ (1.15)	3.14 ^b^ (1.28)
Looking at social media	2.28 (1.38)	2.63 ^a^ (1.42)	1.91 ^b^ (1.25)	2.34 ^a^ (1.38)	1.89 ^b^ (1.15)	2.63 ^c^ (1.50)	2.29 ^a^ (1.35)	2.05 ^a^ (1.26)	2.18 ^a^ (1.42)	2.77 ^b^ (1.48)	1.69 ^a^ (1.03)	2.65 ^b^ (1.45)
Getting ready for bed	2.31 (1.42)	2.64 ^a^ (1.46)	1.96 ^b^ (1.29)	2.36 ^a^ (1.41)	1.87 ^b^ (1.17)	2.70 ^c^ (1.53)	2.38 ^a^ (1.46)	2.07 ^b^ (1.31)	2.08 ^ab^ (1.33)	2.88 ^c^ (1.51)	1.69 ^a^ (1.09)	2.70 ^b^ (1.46)
**Adolescent report**												
Modes of Communication												
In-person conversation	2.74 (1.33)	2.76 ^a^ (1.31)	2.73 ^a^ (1.34)	2.71 ^ab^ (1.33)	2.64 ^a^ (1.31)	2.87 ^b^ (1.30)	2.90 ^ab^ (1.30)	2.65 ^a^ (1.33)	2.61 ^a^ (1.29)	3.00 ^b^ (1.32)	2.40 ^a^ (1.28)	3.03 ^b^ (1.30)
Text messages	1.85 (1.19)	1.93 ^a^ (1.27)	1.79 ^a^ (1.12)	1.83 ^a^ (1.19)	1.83 ^a^ (1.15)	1.97 ^a^ (1.26)	1.94 ^ab^ (1.22)	1.75 ^a^ (1.11)	1.84 ^ab ^ (1.20)	2.04 ^b^ (1.29)	1.54 ^a^ (.97)	2.11 ^b^ (1.29)
Social media	1.62 (1.10)	1.77 ^a^ (1.20)	1.52 ^b^ (1.01)	1.63 ^ab^ (1.12)	1.58 ^a^ (1.05)	1.77 ^b^ (1.19)	1.69 ^ab^ (1.17)	1.57 ^a^ (1.04)	1.60 ^ab^ (1.09)	1.73 ^b^ (1.20)	1.38 ^a^ (.84)	1.83 ^b^ (1.24)
Indirectly	1.68 (1.14)	1.81 ^a^ (1.22)	1.59 ^b^ (1.07)	2.71 ^ab^ (1.33)	2.64 ^a^ (1.31)	2.87 ^b^ (1.30)	1.74 ^ab^ (1.18)	1.62 ^a^ (1.07)	1.66 ^ab^ (1.10)	1.81 ^b^ (1.27)	1.42 ^a^ (.88)	1.91 ^b^ (1.28)
Context of Communication												
Dinner table	1.90 (1.16)	2.05 ^a^ (1.21)	1.80 ^b^ (1.12)	1.88 ^a^ (1.17)	1.82 ^a^ (1.12)	2.08 ^b^ (1.21)	2.00 ^ab^ (1.29)	1.80 ^a^ (1.09)	1.86 ^a^ (1.11)	2.12 ^b^ (1.28)	1.64 ^a^ (.98)	2.12 ^b^ (1.26)
Clothes shopping	2.54 (1.27)	2.45 ^a^ (1.25)	2.61 ^b^ (1.28)	2.50 ^a^ (1.24)	2.58 ^a^ (1.30)	2.67 ^a^ (1.28)	2.42 ^a^ (1.27)	2.45 ^a^ (1.23)	2.49 ^a^ (1.25)	2.81 ^b^ (1.32)	2.20 ^a^ (1.16)	2.83 ^b^ (1.29)
Watching TV	1.84 (1.16)	1.97 ^a^ (1.23)	1.75 ^b^ (1.10)	1.84 ^a^ (1.18)	1.81 ^a^ (1.11)	1.98 ^a^ (1.23)	1.82 ^ab^ (1.16)	1.76 ^a^ (1.11)	1.81 ^a^ (1.09)	2.05 ^b^ (1.27)	1.53 ^a^ (.93)	2.12 ^b^ (1.27)
Grocery shopping	2.00 (1.21)	2.09 ^a^ (1.25)	1.93 ^b^ (1.18)	1.95 ^a^ (1.20)	2.03 ^ab^ (1.23)	2.15 ^b^ (1.25)	2.00 ^ab^ (1.30)	1.86 ^a^ (1.11)	2.00 ^a^ (1.18)	2.27 ^b^ (1.35)	1.65 ^a^ (.99)	2.29 ^b^ (1.30)
Talking about healthy lifestyle	2.52 (1.30)	2.56 ^a^ (1.31)	2.49 ^a^ (1.30)	2.45 ^a^ (1.30)	2.47 ^ab^ (1.33)	2.67 ^b^ (1.26)	2.41 ^a^ (1.25)	2.37 ^a^ (1.25)	2.43 ^a^ (1.23)	2.89 ^b^ (1.39)	2.17 ^a^ (1.21)	2.82 ^b^ (1.30)
Doctor’s office	2.73 (1.29)	2.76 ^a^ (1.28)	2.71 ^a^ (1.29)	2.66 ^a^ (1.27)	2.68 ^a^ (1.30)	2.92 ^b^ (1.28)	2.74 ^ab^ (1.32)	2.59 ^a^ (1.24)	2.67 ^a^ (1.26)	3.08 ^b^ (1.34)	2.41 ^a^ (1.20)	3.01 ^b^ (1.29)
^ ^ Talking about sports	2.12 (1.29)	2.31 ^a^ (1.31)	1.99 ^b^ (1.26)	2.06 ^a^ (1.31)	2.14 ^a^ (1.30)	2.23 ^a^ (1.29)	2.08 ^a b^ (1.24)	2.02 ^a^ (1.25)	2.21 ^ab^ (1.28)	2.28 ^b^ (1.37)	1.84 ^a^ (1.17)	2.35 ^b^ (1.35)
Looking at social media	1.90 (1.25)	1.90 ^a^ (1.26)	1.90 ^a^ (1.25)	1.85 ^a^ (1.21)	1.95 ^a^ (1.32)	2.03 ^a^ (1.31)	2.02 ^a b^ (1.32)	1.82 ^a^ (1.19)	1.93 ^ab^ (1.26)	2.03 ^b^ (1.35)	1.59 ^a^ (1.03)	2.17 ^b^ (1.35)
Getting ready for bed	1.72 (1.14)	1.85 ^a^ (1.18)	1.64 ^b^ (1.09)	1.71 ^a^ (1.12)	1.73 ^a^ (1.17)	1.79 ^a^ (1.15)	1.94 ^a^ (1.28)	1.64 ^b^ (1.08)	1.71 ^ab^ (1.07)	1.87 ^a^ (1.25)	1.41 ^a^ (.85)	1.99 ^b^ (1.27)

Note. Values within the same row and subgrouping not sharing the same letter are significantly different from each other at *p* < 0.01.

## Data Availability

The data in this study are available upon request from the corresponding author, subject to approval from the author’s institutional review board.
